# Subacute combined spinal cord degeneration and pancytopenia secondary to severe vitamin B12 deficiency

**DOI:** 10.1590/S1516-31802012000400010

**Published:** 2012-09-04

**Authors:** José Luis Cabrerizo-García, Mariano Sebastián-Royo, Nerea Montes, Begoña Zalba-Etayo

**Affiliations:** I MD, PhD. Internal Medicine Physician, Internal Medicine Service, Defense General Hospital. Zaragoza, Spain.; II MD. Internal Medicine Physician, Internal Medicine Service, Defense General Hospital, Zaragoza, Spain.; III MD. Intensive Care Physician, Intensive Care Service, Defense General Hospital, Zaragoza, Spain.; IV MD, PhD. Intensive Care Physician, Intensive Care Service, “Lozano Blesa” University Clinical Hospital, Zaragoza, Spain.

**Keywords:** Vitamin B 12 deficiency, Subacute combined degeneration, Pancytopenia, Digestive system abnormalities, Nervous system diseases

## Abstract

**CONTEXT:**

Decreased vitamin B12 concentration does not usually result in clinical or hematological abnormalities. Subacute combined spinal cord degeneration and pancytopenia are two serious and rarely displayed consequences that appear in severe deficits.

**CASE REPORT:**

We present the case of a patient with subacute combined spinal cord degeneration and pancytopenia secondary to severe and sustained vitamin B12 deficiency. Such cases are rare nowadays and have potentially fatal consequences.

**CONCLUSIONS:**

Vitamin B12 deficiency should be taken into consideration in the differential diagnosis in cases of blood disorders or severe neurological symptoms. Early diagnosis and treatment can avoid irreversible consequences.

## RESUMEN

**CONTEXTO:**

El descenso de la concentración de vitamina B12 no suele derivar en alteraciones clínicas o hematológicas. La degeneración combinada subaguda medular y la pancitopenia son dos consecuencias graves y poco frecuentes que aparecen en los déficit severos.

**CASO CLÍNICO:**

Presentamos el caso de un paciente con una degeneración combinada subaguda medular y pancitopenia secundarios a un déficit severo y mantenido de vitamina B12. Un caso poco común en nuestros días y con consecuencias potencialmente fatales.

**CONCLUSIONES:**

Debemos considerar el déficit de vitamina B12 dentro del diagnóstico diferencial en caso de alteraciones hematológicas o síntomas neurológicos graves. Su diagnóstico y tratamento precoz pueden evitar secuelas irreversibles.

## INTRODUCTION

Low serum vitamin B12 levels are common, especially in older people. Vitamin B12 is obtained through animal products consumed in the diet. Vitamin B12 deficiency is usually a response to insufficient intake or gastrointestinal disorders. Because of the low requirements in humans (2.4 mg/day), clinical or subclinical deficit may be delayed. Vitamin B12 deficiency can lead to blood disorders, nervous system disorders or neuropsychiatric conditions. However, the clinical picture does not always accompany the analytical variations.^1^ Correction of the deficit is important, especially in severe cases, and neurological sequelae may be irreversible if treatment is not started in good time.

## CASE REPORT

We present the case of a 59-year-old patient without allergies or toxic habits, who was a welder by profession, with a history of hypertension and dyspepsia. His diet was varied.

His treatment had been omeprazole, plantago ovata and aspirin for years. He started to experience tingling in both arms one year before the time of this report, and it went on gradually increasing. Six months later, he presented difficulty in walking, feelings of instability and decreased strength in all four limbs, especially the lower limbs. He had been losing the flavor of foods and, in the last month, he presented severe fatigue and rapidly progressive cognitive impairment with memory problems, with episodes of agitation, confusion and incoherent speech. Over the days before going to the hospital, he presented total inability to move the lower extremities and a significant decrease in upper-limb strength, with numbness in both the arms and the legs.

**Figure 1.**
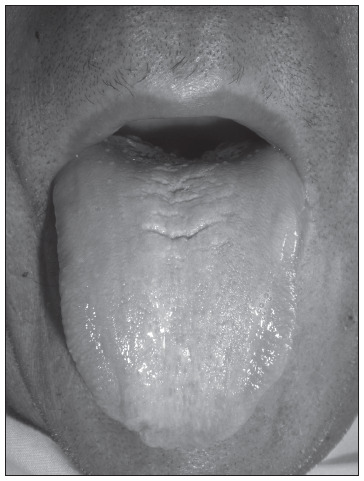
Atrophic glossitis.

At the hospital, we observed intense pallor, depressed level of consciousness (Glasgow 9), slurred speech, blood pressure of 80/40 mmHg and heart rate 110 bpm. The neurological examination showed absence of deep sensitivity (positional and vibratory), absence of the sense of touch in the lower limbs, motor deficit of 2/5 in the right leg and 1/5 in the left leg, patellar and Achilles tendon reflexes absent, and left extensor Babinski reflex present. The upper extremity motor deficit was 3/5, with diminished tactile sensitivity and deep tendon reflexes. The tongue had a bright red depapillated appearance (Figure 1). Among the analytical parameters, the following were observed (normal values are in brackets): hemoglobin: 3.3 g/dl (12-18); hematocrit: 10.1% (37-52); erythrocytes: 850,000 cell/ml (4,200-6,100); mean corpuscular volume: 114.8 fl (76-96); mean corpuscular hemoglobin: 37.5 pg (27-32); red cell distribution width: 19.1% (11.5 to 14.5); reticulocytes: 1.74% (0.5-2); leukocytes: 3,030 cell/mm^3^ (4,800-10,800), platelets: 63,000 cell/mm^3^ (130,000-400,000), indirect bilirubin: 2.12 mg/dl (< 0.7) and lactic dehydrogenase: 9,307 U/l (160-480). The patient was admitted to the intensive care unit where four packed red blood cell units were transfused and fluids were administered until clinical stabilization was achieved. A vitamin B12 test was requested, and the concentration found was 30 pg/ml (157-1050), while the folate concentration was 9.1 ng/ml (2.76-18.2). Thyroid stimulating hormone and tyrosine T4 concentrations were normal.

**Figure 2.**
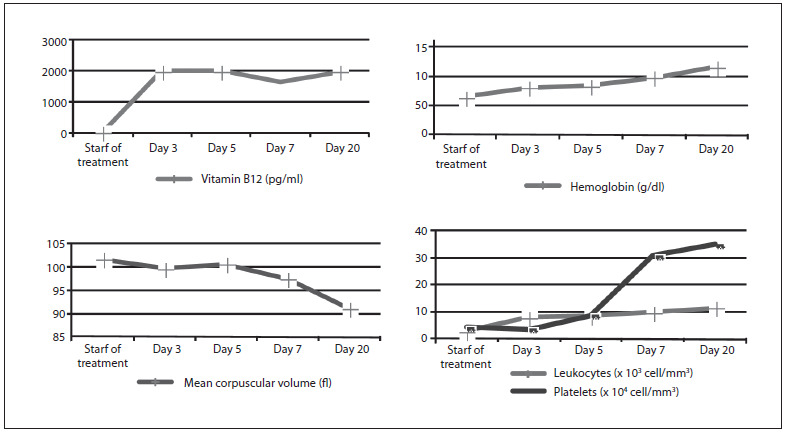
Evolution of hematological parameters after start of treatment with vitamin B12.

Vitamin B12 treatment was started intramuscularly at a dose of 1,000 mcg/day, and with oral administration of B12 complex (0.5 mg) + B6 (250 mg) + B1 (250 mg) twice a day. Gastroscopy with biopsy showed a sliding hiatal hernia, chronic superficial gastritis and severe atrophy with intestinal metaplasia in the antral mucosa. The remaining malabsorption, serological, radiological and immunological evaluations were negative or normal. After several days of treatment, bone marrow aspiration showed red and white cell hyperplasia with significant megaloblastosis, consistent with vitamin B12 deficiency in the patient, in response to the treatment phase, without other dysplastic signs. Figure 2 shows the evolution of different parameters after starting the treatment with vitamin B12.

Within the first week, the patient’s cognitive status was fully restored to normal and the vitamin B12 dose was changed to 1,000 mcg/week, intramuscularly. After the first month of treatment and early rehabilitation, the patient showed improvement in the lower limb motor deficit with recovery to 4/5 bilateral tactile sensitivity, but without recovering deep sensitivity, he tolerated sitting but not walking. The paresthesia in the legs and ageusia had improved substantially. We continued the intramuscular treatment with 1,000 mcg/month and the patient then moved to another hospital for further specific rehabilitation treatment. The final outcome was favorable, but the patient was still unable to fully recover his deep sensitivity.

## DISCUSSION

Pancytopenia caused by vitamin B12 (cobalamin) deficiency is rare and reflects advanced stages of severe deficits that are sustained over time. The most common causes of vitamin B12 deficiency include pernicious anemia, gastritis, impaired absorption secondary to surgery or inflammatory bowel disease, pancreatic insufficiency, vegan diets, drugs that block absorption such as inhibitors of proton pump inhibitor, biguanide neomycin, anesthesia with nitric oxide or genetic disorders such as deficiency of transcobalamin II. Our case had two of these possible etiologies: chronic atrophic gastritis and treatment with omeprazole for a long time.

The most common hematological abnormality is macrocytic anemia with elevated lactic dehydrogenase secondary to ineffective erythropoiesis, with a normal or slightly decreased reticulocyte count. The bone marrow is hypercellular with megaloblastic erythroid hyperplasia and giant metamyelocytes. In addition to the symptoms resulting from reduction of the hematological series, vitamin B12 deficiency may cause damage to different levels of the nervous system secondary to demyelination with vacuolar degeneration and gliosis. This may result in myelopathy, peripheral neuropathy, cognitive impairment and optic atrophy. However, not all patients with neurological disorders present hematological abnormalities. The myelopathy thus caused is called subacute combined degeneration. The posterior or posterolateral column is the part most affected, especially at the cervicodorsal level. The clinical manifestations range from impaired positional sense and vibration up to spastic paresis or tetraparesis.^2^ The clinical sequence usually consists initially of general weakness and numbness in the hands and feet, followed by unsteady gait, stiffness and weakness of the lower extremities.^2^

Subsequently, our patient had symptoms of posterior and lateral column involvement, with loss of vibratory and positional sensitivity that started in the lower limbs and extended outwards into the trunk and upper extremities. Motor signs such as loss of strength, spasticity, changes in tendon reflexes, clonus and extensor plantar response presented delayed onset. Patellar and Achilles reflexes are affected first, and may become increased, decreased or even absent. Involvement is usually symmetrical. There may be irritability, apathy, drowsiness, confusion, depressive syndrome, dementia and visual impairment through optic nerve involvement in advanced cases. The basis of the diagnosis is physical examination and laboratory findings. When the values of vitamin B12 go below 100 pg/ml, neurological symptoms usually appear. However, there is not always any parallel between them.^3^ Methylmalonic acid and homocysteine are metabolites of cobalamin, and their levels increase when the tissue reserve drops, thereby more closely reflecting the vitamin B12 deficit.^3^

Electroneurogram recordings provide an additional diagnostic test, in which there is a marked slowing of sensory conduction or somatosensory evoked potentials. Magnetic resonance with T2 hyperintense imaging may reflect demyelination of the posterior column, most frequently at the cervical and thoracic levels.^4^

The traditional treatment for vitamin B12 deficiency consists of intramuscular doses of 1,000 mcg/day for a week, then 1,000 mcg/week for a month and, subsequently, 1,000 mcg/month for life. This avoids involvement of the intrinsic factor produced by parietal cells and its absorption in the terminal ileum. However, it is known that 1% of passively ingested vitamin B12 is absorbed without participation by the intrinsic factor, thus enabling oral treatment with high doses and hematological and neurological response rates that are similar over the short-term, while lowering the cost and being better tolerated.^5^^,^^6^ The hematological parameters in our case improved rapidly after treatment was started.

The clinical response is inversely proportional to the magnitude and duration of the disease.^7^ Recovery may be complete if symptoms have only been present for a few weeks before the start of treatment. If the interval has been longer, the best that can be hoped for is containment of the symptoms.^8^

We made a search for similar cases in different databases (Pubmed, Lilacs, Embase and Scirus) using the terms: “Subacute combined spinal cord degeneration” AND “pancytopenia” AND “vitamin B12 deficiency”. The result was only three items in Pubmed (Table 1).

**Table 1.** The results from searches in the medical databases using the descriptors of the clinical findings from our patient. Date of search: April 13, 2011

Database	Search strategy (descriptors)	Results^9^^,^^10^^,^^11^	Related articles
Lilacs	“Subacute combined spinal cord degeneration” AND “pancytopenia” AND “vitamin B12 deficiency”	There were no references in this database	None
PubMed	“Subacute combined spinal cord degeneration” AND “pancytopenia” AND “vitamin B12 deficiency”	3 case reports	Ishiko et al.^9^; Zara et al.^10^; Nagaishi et al.^11^
Embase	“Subacute combined spinal cord degeneration” AND “pancytopenia” AND “vitamin B12 deficiency”	There were no references in this database	None
Scirus	“Subacute combined spinal cord degeneration” AND “pancytopenia” AND “vitamin B12 deficiency”	There were no references in this database	None

## CONCLUSIONS

Vitamin B12 deficiency should be suspected in the presence of elevated mean corpuscular volume with or without anemia; hypersegmented neutrophils; pancytopenia of unknown or unexplained origin; and neurological signs and symptoms like dementia, progressive weakness, ataxia or paresthesia.

All patients with vitamin B12 deficiency should be investigated to determine its cause and wheter it might be reversible. In all cases, replacement therapy should be administered.

Although oral and parenteral administration appear to be equally effective in patients with severe neurological symptoms, it is preferable to begin the treatment intramuscularly to ensure compliance.
